# The European Health Union and the protection of public health in the European Union: Is the European Union prepared for future cross-border health threats?

**DOI:** 10.1007/s12027-023-00732-1

**Published:** 2023-04-18

**Authors:** Claudia Seitz

**Affiliations:** 1 Private University in the Principality of Liechtenstein, Triesen, Liechtenstein; 2 University of Ghent, Ghent, Belgium; 3 University of Basel, Basel, Switzerland; 4 University of Bonn, Bonn, Germany

**Keywords:** Health law and policy, Covid 19 pandemic, European Health Union, Health Security Committee (HSC), European Centre for Disease Prevention and Control (ECDC), EU Health Emergency Preparedness and Response Authority (HERA)

## Abstract

The Covid 19 pandemic has cast traditional health protection issues in a new light due to their cross-border significance with far-reaching consequences for almost all areas of social life and places health protection in the European Union in a broader context that goes beyond the national consideration of necessary measures at EU Member State level. The pandemic has made it clear that the responsibility for public health remains in principle with the EU Member States and that the competences of the European Union under Article 168 TFEU are – with a few exceptions – generally limited to supporting, coordinating and assisting tasks. This article examines whether the European Union is adequately prepared for future pandemics and other cross-border health threats based on its responsibilities under the current system of competences between the EU and its Member States in the area of health policy under article 168. The article concludes with some suggestions for discussion and consideration.

## Introduction

Covid 19 changed the lives of individuals and the society to an unprecedented extent.^1^ In addition to numerous restrictions on the individual’s fundamental rights and the justification of these restrictions by reasons based primarily on the idea of solidarity towards vulnerable groups of society, the pandemic led not only to far-reaching consequences for the individual, but also to fundamental changes at the societal level. In addition to the power of the executive branch within the state, justified by emergency measures, the structure of competences in health policy between the European Union (EU) and its Member States during a global health crisis became visible at the European level through the example of the Covid 19 pandemic. This structure was largely shaped by nation-state measures of the Member States to protect (national) public health and partly revealed the powerlessness of the EU in various areas of health protection. This was conditioned by the existing competence structure between the EU and its Member States.

How fundamental these developments were cannot be conclusively assessed even now. Nevertheless, due to its far-reaching significance for the protection of life and health and the socio-economic consequences^2^ resulting from the necessary protective measures,^3^ the Covid 19 pandemic, which was already described as a “once-in-a-century event” as early as the beginning of the pandemic,^4^ can be regarded as a turning point in multiple sectors of society, which also makes a (re)consideration of the protection of public health necessary in future pandemics. Against this background, the Covid 19 pandemic puts traditional health protection issues – due to their worldwide significance with far-reaching consequences for almost all areas of social life – in a new context and places them in a broader correlation in light of global causes and effects.^5^

At the European level, the questions that arise with regard to health protection are, in particular, whether the EU and the Member States are sufficiently prepared for future cross-border health crises and can adequately ensure the protection of the health of EU citizens. In this context, further questions can be raised in light of the experience with the Covid 19 pandemic, such as in particular whether the measures taken at EU level during the Covid 19 pandemic were sufficient and appropriate, whether further health protection measures should have been taken at EU level rather than at a national level, and whether the system of competences between the EU and the Member States should be reconsidered with regard to cross-border health threats, especially in the case of pandemics. In addition to the initiatives and measures already initiated by the EU Commission during the Covid 19 pandemic, questions also arose as to whether the European Health Union with its function and tasks, as well as the establishment of a new authority, represent a sufficient response to future cross-border health threats in the EU, or whether the existing structure of competences should not rather be reconsidered – in addition to these measures – at least with regard to pandemics that could cause a health emergency of international concern as defined by the World Health Organization (WHO) as a “Public Health Emergency of International Concern” (PHEIC)^6^ according to the 2005 International Health Regulations (2005 IHR).^7^

The following article will explore these questions and place them in a broader context. To this end, the current distribution of competences between the EU and the Member States for the protection of the public health will first be analysed and presented. Based on the different concepts and systems of health policy of the Member State, the common basic values of health policy will be presented, which constitute the current system of the distribution of competences between the EU and the Member States for the protection of public health. Building on this, the Covid 19 pandemic in the EU and the measures taken in this context will be briefly discussed, in which the existing EU institutions – the Health Security Committee (HSC) and the European Centre for Disease Prevention and Control (ECDC) – will be assessed. This will be followed by a brief analysis of the European Health Union, outlining the coordination measures, preparedness and risk planning, and the new EU Health Emergency Preparedness and Response Authority, before examining the question of whether there is a need to extend the competences of the EU in pandemic cases which are so serious that they constitute a public health emergency of international concern and require increased joint action by the Member States, before summarising and assessing the outcome.

## Health protection at the EU level

### Ensuring a high level of health protection

#### Differences in health policy in the EU and common values

The design of health policy within the EU is strongly influenced by the different health care systems in the individual Member States, which sometimes differ considerably in their concept and financing.^8^ These differences not only affect the different levels of national social protection, such as health insurance and reimbursement of services,^9^ but also the legal framework of many other areas of health law, such as state measures to protect against infection, as became evident during the Covid 19 pandemic.^10^ The respective design of national health policy is based on a variety of factors, some of which have evolved historically, and reflects different socio-political decisions with regard to health protection.

Despite differences in design, organisation and financing, the national health systems of the Member States are based on common values, such as guaranteeing good quality health care and equal treatment with regard to access to such health care. Regarding the fundamental values of health protection within the EU, the Council of the EU had already stated in 2006 that the health systems “are a central part of Europe’s high levels of social protection” and make a “major contribution to social cohesion and social justice”.^11^ The “over-arching values of universality, access to good quality care, equity, and solidarity” would be widely accepted in the work of the different EU institutions and would constitute “a set of values that are shared across Europe”.^12^

The protection of health within the framework of health policy design thus represents a particularly sensitive policy area of the EU due to the different traditions and structures of the Member States’ health systems, diverging national priorities and values as well as different financing concepts of the health sector. Although the Member States are fundamentally responsible for their health policies, the design of national health care systems and health protection in general at the national level, the above-mentioned areas are based on common values.

#### Emergence of an EU health policy

The Treaty of Maastricht of 1992 establishing the EU^13^ created a legal basis for the introduction of health policy measures at the EU level by incorporating health protection. This was the first time that health protection was enshrined at EU level.^14^ Health protection was further strengthened in the Treaty of Amsterdam of 1997 by the introduction of new provisions, according to which the EU could for the first time adopt measures with the aim of ensuring a “high level of human health protection” and was not limited – as before – to merely contributing to these.^15^ Art. 129 (1) of the Treaty of Amsterdam states: “A high level of human health protection shall be ensured in the definition and implementation of all Community policies and activities. Community action, which shall complement national policies, shall be directed towards improving public health, preventing human illness and diseases, and obviating sources of danger to human health”.^16^

For the first time, however, health policy at the EU level gained in importance through the Treaty of Lisbon of 2007.^17^ Against the background of the differences in the health systems of the Member States and the different forms of national health protection, Art. 2 C (2) lit. k of the Treaty of Lisbon assigns the EU shared competence in this particularly sensitive policy area with regard to “common safety concerns in public health matters” concerning the aspects mentioned in the Treaty of Lisbon.^18^ Thus, the Treaty of Lisbon extended the legislative powers of the EU Parliament to the area of “public health”. Nevertheless, no comprehensive competences in the area of health protection or health policy were transferred to the EU. Rather, the EU basically only has a coordinating and supporting function in the area of health protection. Art. 2 E p. 1 of the Treaty of Lisbon states that the Union “shall have competence to carry out actions to support, coordinate or supplement the actions of the Member States”, whereby these measures can be taken “at European level” in the area of “protection and improvement of human health” (Art. 2 E p. 2 lit. a of the Treaty of Lisbon).

#### High level of health protection in EU policies and measures

At the EU level, health policy gained importance through the Treaty on the Functioning of the European Union (TFEU):^19^ Art. 168 TFEU regulates the Union’s contribution to ensuring human health protection in the EU. According to Art. 168 (1) TFEU Union action, “which shall complement national policies” shall be “directed towards improving public health, preventing physical and mental illness and diseases, and obviating sources of danger to physical and mental health”.^20^ In addition, Art. 169 TFEU covers consumer protection. Both primary law provisions show the basic decision of the Treaties to create and maintain high standards of protection in the areas of health and consumer protection.^21^ Likewise, both provisions form a separate title in Part Three of the TFEU on the “Union policies and internal actions”. Health and consumer protection can thus not only be co-regulated as an annex to other areas, but can be made the main subject of EU measures themselves. However, this does not exclude that they can also be the subject of harmonisation measures within the framework of the internal market provisions according to Art. 114 TFEU.

The central provision with regard to health protection is Art. 168 (1) TFEU: “A high level of human health protection shall be ensured in the definition and implementation of all Union policies and activities”. Although Art. 168 TFEU is under the heading “Title XIV Public Health”,^22^ it does not refer to “health” per se, but to health protection activities.^23^ The provision aims at protecting the health of the population and thus at public health and not primarily at individual health.^24^ The objective of health protection according to Art. 168 TFEU is thus clearly directed towards the health of the population and thus towards the protection of public health.

Health protection was further strengthened at the level of the Charter of Fundamental Rights of the European Union (CFR) of 2000,^25^ as Art. 35 CFR establishes principles of health protection. According to Art. 35 p. 1 CFR, “[e]veryone has the right of access to preventive health care and the right to benefit from medical treatment under the conditions established by national laws and practices”. Although this provision does neither establish a right to health nor a right to protection as such, it does, however, constitute a “right of access to preventive health care”, but limits this right again by restricting it to the extent permitted by national law and practice. Furthermore, Art. 35 p. 2 CFR states: “A high level of human health protection shall be ensured in the definition and implementation of all Union policies and activities”. Art. 168 (1) TFEU is therefore in principle identical to Art. 35 p. 2 CFR,^26^ except for editorial differences.^27^

### System of competences in the area of health policy

#### General principles of the allocation of competences to the EU

With regard to the allocation of competences in Art. 168 TFEU and the subsequent classification and evaluation of the European Health Union, the general principles of the allocation of competences to the EU shall be briefly examined: According to Art. 5 (1) and (2) TEU, the general principle for the delimitation of competences is the “principle of conferral”, according to which the EU shall act only within the limits of the limits of the competences conferred upon it in primary law and in accordance with the powers assigned therein to the EU institutions.^28^ This establishes that the EU only has enumerative competences and not a general competence.^29^

The principles of subsidiarity and proportionality apply to the exercise of the Union’s competences (Art. 5 (1) sentence 2 TEU).^30^ According to the principle of conferral, the EU “shall act only within the limits of the competences conferred upon it by the Member States in the Treaties to attain the objectives set out therein” (Art. 5 (1) sentence 1 TEU). Competences not conferred upon the EU in the Treaties remain with the Member States (Art. 5 (2) TEU). In the area of health protection, competences have been transferred to the EU; according to the general principles under Art. 5 TEU, all competences not transferred remain with the Member States.

#### Competences of the EU in the area of health policy

The EU’s health policy has been given a legal basis through Art. 168 TFEU and has thus gained in importance. Although the Member States are still fundamentally responsible for health protection and, in particular, health care systems, limited competences have been transferred to the EU, so that the EU has its own scope for action in the areas of public health and health protection within the framework of the competences assigned to it in Art. 168 TFEU. In general, however, the competence for the organisation of health services and medical care still lies with the Member States. Art. 168 (5) TFEU even explicitly prohibits the EU Parliament and the Council to take measures in the mentioned areas to harmonise the laws and regulations of the Member States.

At first glance, the areas of competence under Art. 168 TFEU seem complex and multi-layered: The provision covers horizontal as well as vertical tasks in the area of health policy, contains competences and provides for certain forms of action as well as decision-making procedures.^31^ The scope of application is defined in terms of objectives, whereby the objectives are primarily oriented towards preventive health protection.

However, this does not exclude aspects of curative or rehabilitative medicine as far as the improvement of the public health, the prevention of human diseases and the elimination of sources of danger to physical and mental health are concerned.^32^ The EU “complements” the actions of the Member States or “encourages” the cooperation of the Member States in principle, so that it basically has a supporting function in the area of health protection.^33^

The EU’s scope for action under Art. 168 TFEU primarily concerns the areas of health promotion and prevention and here, among other things, actions to eliminate sources of danger to human health or to combat widespread serious diseases. The areas of activity in which the EU exercises a supporting role include, in particular, the improvement of public health and the prevention of human illness (Art. 168 (1) subpara. 2 p. 1 TFEU), the fight against the major health scourges (Art. 168 (1) subpara. 2 p. 2 TFEU), the monitoring, early warning and combating of serious cross-border threats to health (Art. 168 (1) subpara. 2 p. 2 TFEU) as well as early warning of and combating serious cross-border threats to health and measures which have as their direct objective the protection of public health regarding tobacco consumption and alcohol abuse (Art. 168 (5) TFEU).

This illustrates that Art. 168 TFEU lists concrete health policy fields of action in various passages.^34^ The list of fields of action, some of which are difficult to distinguish from each other or appear redundant, is merely a prioritisation, so that a precise delimitation of the fields of action mentioned is not absolutely necessary. The EU is only given its own competences in the narrowly defined exceptions in Art. 168 (4) TFEU. Accordingly, the EU Parliament and the Council contribute to the achievement of the objectives listed in the following catalogue of tasks, such as measures setting high standards of quality and safety of organs and substances of human origin, blood and blood derivatives (lit. a), measures in the veterinary and phytosanitary fields which have as their direct objective the protection of public health (lit. b) and measures setting high standards of quality and safety for medicinal products and devices for medical use (lit. c).

#### Competences of the Member States in the area of health policy

Art. 168 (7) TFEU provides that Union action shall respect the responsibilities of the Member States for the definition of their health policy Art. 168 (7) p. 1 TFEU stipulates that the “responsibilities of the Member States for the definition of their health policy and for the organisation and delivery of health services and medical care” shall be respected. According to Art. 168 (7) p. 1 TFEU the “Union action shall respect the responsibilities of the Member States for the definition of their health policy and for the organisation and delivery of health services and medical care”. Pursuant to Art. 168 (7) p. 2 TFEU, the “responsibilities of the Member States shall include the management of health services and medical care and the allocation of the resources assigned to them”. Thus, direct harmonisation measures by the EU are excluded, with the exceptions mentioned, so that the EU can in principle only act in a complementary coordinating or supporting capacity.^35^

Overall, the competences listed in Art. 168 TFEU are largely focused on health-related prevention and emergency response law and not on the health systems of the Member States.^36^ These are supplemented by various internal market and budgetary requirements for the design of national health systems: While the EU has only limited competences based on Art. 168 TFEU, its influence on the national health systems of the Member States is evident in connection with the fundamental freedoms in the internal market. Based on Art. 114 TFEU and with the objectives, in particular, of the free movement of goods and the freedom to provide services, goods and services related to health are regulated at the EU level. The regulations cover goods, such as food, cosmetics and tobacco products, as well as services, e.g. in the context of patient mobility.

However, it is clear from the system and, in particular, from the provision under Art. 168 (7) TFEU that the responsibilities for health protection remain fundamentally within the competence of the Member States, as Art. 168 TFEU does not confer any comprehensive or far-reaching competences on the EU in this regard. The exceptions to the prohibition of harmonisation under Art. 168 (7) TFEU only apply to the areas explicitly listed in Art. 168 (4) TFEU, which concern measures that address “common safety concerns”.^37^ Only in these narrowly defined areas does the EU have full shared legislative competence, such as measures setting high quality and safety standards for medicinal products and medical devices (Art. 168 (4)(c) TFEU). The basic principle of European health policy is thus a complementary European competence, according to which the EU supports the national health policies of the Member States, but may not pursue its own objectives in doing so.

Consequently, on the basis of the principle of subsidiarity according to Art. 5 (3) TEU, the EU, in areas that do not fall within its exclusive competence, shall act only if and in so far as the objectives of the proposed action cannot be sufficiently achieved by the Member States, either at central level or at regional or local level, but can rather, by reason of the scale or effects of the proposed action, be better achieved at Union level. In accordance with the requirements of the principle of subsidiarity, according to Art. 4 (2) (k) TFEU, “common safety concerns in public health matters for the aspects defined to in this Treaty” fall within the “shared competence” between the EU and the Member States.^38^ However, the shared competence only refers to the area of “common safety concerns”, so that other areas related to health protection remain within the competence of the Member States.^39^ Thus, EU law leaves the competences of the Member States to organise the respective national health system – except for the areas mentioned – basically unaffected.^40^

Furthermore, Art. 153 (4) TFEU explicitly states with regard to the organisation of health care systems that the EU has no competences in the area of social security systems, as the right of the Member States to define the fundamental principles of their social security systems must not be affected.^41^ Accordingly, all regulations and decisions that affect a central area of the national competence to govern the health system remain within the competence of the Member States, as this subject area traditionally falls under the functional guarantee for national governance decisions of the Member States according to Art. 168 (7) TFEU.^42^ Overall, the EU therefore has no general competence to pursue autonomous health policy or to influence or even harmonise the health systems of the Member States. Health policy, health care and thus also the organisation of health protection are therefore – with the exceptions mentioned – fundamentally within the competence of the Member States.^43^

## Covid 19 pandemic

### EU health protection during the Covid 19 pandemic

The Covid 19 pandemic and its aftermath have highlighted the difficulties, challenges and hurdles faced by the EU in supporting Member States to coordinate responses and actions in the face of a cross-border health crisis.^44^ At the beginning of the pandemic, EU coordination was often criticised as being too slow, for example, in assessing the prevalence of Covid 19 infections or the approval of vaccines by the European Medicines Agency (EMA).^45^ However, the delays for which the EU was criticized in responding to the Covid 19 pandemic are based on the system of competences outlined and the limited allocation of competences to the EU within the framework of Art. 168 TFEU. Some of the shortcomings of the EU that became apparent during the Covid 19 pandemic reflect – at least in part – the limits of competences that the EU has in the areas of health policy, public health and health protection under Art. 168 TFEU.

Accordingly, during the Covid 19 pandemic, the EU was in its actions largely dependent upon and limited to the support of the Member States. The EU Commission, for example, coordinated the “joint Corona crisis response”, supported the Member States in coordinating their national measures, and provided information on the spread of the virus and effective measures to contain it.^46^ Nevertheless, specialized institutions – also for the prevention and control of diseases – had already been established before the Covid 19 pandemic and were also active during the pandemic, which will be briefly discussed below.

### Health Security Committee (HSC)

The prevention of infectious diseases has been an early priority for the EU, requiring a global and coordinated approach among Member States. To achieve this priority, Decision No 2119/98/EC setting up a network for the epidemiological surveillance and control of communicable diseases established a system in 1998 to serve as an early warning and response system for the prevention and control of communicable diseases, in addition to epidemiological surveillance.^47^ The Decision provided for epidemiological surveillance through the systematic and continuous collection, analysis, evaluation and dissemination of health data, including epidemiological studies.^48^

Decision No. 2119/98/EC setting up a network for the epidemiological surveillance and control of communicable diseases in the community was repealed by Decision No. 1082/2013/EU on serious cross-border threats to health.^49^ This decision formalised and strengthened the rate of the Health Security Committee (HSC), which was setup in the aftermath of the 2001 terrorist attacks in the USA.^50^ Its task is to provide a coherent and well-coordinated response to cross-border health threats. The HSC coordinates EU health security measures in general and is not limited to communicable diseases. The Committee has laid the groundwork for various preparedness activities. These include, in particular, enabling Member State governments to share information and assess health events, as well as serving as a discussion forum to advise health ministers and facilitate coordinated crisis response by Member States.

The Committee is composed of representatives from each national administration, the Commission’s Directorate–General for Health and Consumers, and other Commission services and agencies (e.g. the European Centre for Disease Prevention and Control and the European Medicines Agency). During the Covid 19 pandemic, coordination was facilitated by the Health Security Committee as the central board for coordinating the response of the EU. Nevertheless, the Member States were able to decide for themselves, based on their competences, whether and which measures were appropriate to prevent, control and combat the Covid 19 pandemic at the national level. Consequently, the EU had only a supporting and thus subordinate role in the context of pandemic control compared to the Member States.^51^

### European Centre for Disease Prevention and Control (ECDC)

Previous threats to public health from infectious diseases have already revealed shortcomings at the EU level even before the Covid 19 pandemic. For example, the spread of SARS in 2002 demonstrated that dealing with cross-border health threats from infectious diseases could not be addressed at the Member State level alone and that an institutionalisation of health risk management at the EU level was needed.

As a result, the European Centre for Disease Prevention and Control (ECDC) was established in 2005.^52^ The ECDC is an EU agency based in Stockholm and was established with the aim of preventing and controlling infectious diseases in the EU.^53^ The establishment of this agency is based on Regulation (EC) No 851/2004 establishing a European Centre for disease prevention and control.^54^ The aim of this centre is to support the EU in identifying risks arising from current and emerging threats to human health from infectious diseases. To this end, the ECDC is to cooperate with the national European health authorities to enable international surveillance of diseases and to develop early warning systems.

The first recital of Regulation (EC) No 851/2004, according to which the Union is committed “to protect and to improve human health by prevention of human disease, in particular communicable diseases, and to counter potential threats to health with a view to ensuring a high level of protection of health of European citizens”, clearly illustrates this. An effective response to disease outbreaks, as the first recital states, “requires a coherent approach among Member States and input from experienced public health experts, coordinated at Community level”. The creation of the ECDC was not based on a change in the existing balance of competences between the Member States and the EU, as the agency did not receive any additional rights or competences from the Member States.^55^ Nevertheless, it led to an institutionalisation of health protection in the prevention and control of infectious diseases in the EU.^56^ Thus, the creation of the ECDC appears as the result of a process and as a complement to the already existing networks for infectious disease surveillance.

While in January 2020 Member States did not yet see the need to coordinate their responses to the Covid 19 pandemic at the EU level, possibly underestimating the impact of the pandemic and the resources required, the pandemic escalated within a very short time and became a major threat to the entire population in the EU.^57^ This rapid escalation became an obstacle to coordination at the EU level, so that the EU Commission’s advisory panel on Covid 19 was not established by the Member States until March 2020.

One consideration in the aftermath of the Covid 19 pandemic was to strengthen the tasks of ECDC.^58^ However, the ECDC’s mandate under Regulation 851/2004 is limited to the surveillance of risks to human health from communicable diseases and explicitly excludes risk management measures. However, public health mechanisms at EU level, such as the ECDC, can only play a more active role if key EU policy actors agree on a common response.^59^

### Covid 19 and public health protection in the EU

The health crisis caused by the Covid 19 pandemic and the threat to public health in the EU from previous infectious diseases, such as SARS or previous influenza infections, were not comparable to the extent and consequences of the Covid 19 pandemic. Thus, the EU Commission emphasises that the pandemic has shown how important coordination between European countries is for health protection.^60^ However, this does not only apply to crises, but also during normal times, which provide opportunities to treat widespread diseases, invest in efficient health systems and train health workers.^61^

In the light of these difficulties and delays, which became apparent in the course of the Covid 19 pandemic, various reflections on new mechanisms, such as in particular the creation of a “European Health Union” as well as a new agency, the EU Health Emergency Preparedness and Response Authority (HERA), have been undertaken. These are presented below and analysed with regard to infection control at EU level.

## European Health Union

### Background and objectives

In order to better address cross-border health threats within the EU, the EU had already planned to establish a “European Health Union” in mid-2020.^62^ The creation of a European Health Union is in particular a response to the challenges of the Covid 19 pandemic.^63^ The importance of the European Health Union for health protection in the EU is made clear by the quote from Commission President *Ursula von der Leyen*: “We cannot wait for the end of the pandemic to repair and prepare for the future. We will build the foundations of a stronger European Health Union in which 27 countries work together to detect, prepare and respond collectively.”^64^

The idea for a European Health Union emerged in the course of the Covid 19 pandemic. In view of the deficits, the European Council called on the EU Commission as early as March 2020 to present reform proposals for a “more ambitious and wide-ranging crisis management system within the EU”.^65^ In a resolution in July 2020, the EU Parliament also called for a European Health Union, which should include common minimum standards for quality healthcare, a European Health Response Mechanism (EHRM) to respond to all types of health crises and a European fund to strengthen hospital infrastructure.^66^ The EU Commission complied with this demand with its communication on the establishment of a European Health Union.

The European Health Union consists of a series of proposals to strengthen existing EU measures and, in particular, to enhance the function and tasks of key EU agencies in crisis preparedness and response. Core elements of the European Health Union include, in particular, the reshaping of the existing legal framework for serious cross-border health threats and the upgrading of the roles and functions of key EU agencies in crisis preparedness and response, notably the European Centre for Disease Prevention and Control (ECDC) and the European Medicines Agency (EMA), as well as the establishment of a new EU authority for emergency preparedness and response to public health crises.

Of fundamental importance in the design of the European Health Union is that it should cover future pandemics, but is not limited to communicable diseases. Rather, the EU Commission wants to build a strong European Health Union in which all Member States of the EU participate in crisis preparedness and management, contribute to available, affordable and innovative medical supplies, and work together to improve the prevention, treatment and aftercare for diseases such as cancer.^67^ In particular, the European Health Union aims to achieve better health protection for EU citizens, European and national capacities to better prevent and manage future pandemics, and more resilient European health systems.^68^ The individual core elements will be briefly outlined and analysed below.

### Coordination measures

As the EU Commission emphasizes in its Communication on the European Health Union, joint efforts to combat the Covid 19 pandemic, as well as other future health crises, require greater coordination at EU level.^69^ Public health measures would need to be coherent and coordinated to maximize their impact and minimise harm to people and businesses.^70^ The health situation of each Member State is dependent on the health situation in the other Member States, with fragmented responses to cross-border health threats making all Member States collectively more vulnerable.^71^ Against this background, the EU Commission wants to build a strong European Health Union in which all Member States participate in health crisis preparedness and management, contribute available, affordable and innovative medical supply, and work together to improve prevention, treatment and aftercare for diseases such as cancer.^72^

The EU’s actions under the European Health Union cover the main areas where the EU can play a coordinating and supporting role and are based on the experience of the Covid 19 pandemic.^73^ In particular, measures such as joint procurement or addressing supply shortages are foreseen. The benefits associated with the European Health Union are, in particular, better health protection, the building of European and national capacities to better prevent and manage future pandemics, and more resilient European health systems.^74^

### Preparednes and response planning

In its Communication on the creation of a European Health Union, the EU Commission points out the previous weaknesses in preparedness and emergency planning: Covid 19 has revealed that preparedness and response capacities at national level had been insufficient, which became apparent when many Member States found that their stockpiling was inadequate, they lacked immediately available surge capacities for health care systems, testing, contact tracing and surveillance, the lack of implementable business continuity plans for healthcare provision, and shortages of qualified medical staff.^75^

In addition, Covid 19 had also revealed that there was a clear lack of an overall vision on the operationalisation of Member State preparedness and response plans, as well as an incoherence with regards to their compatibility.^76^ This was due to the fact that the EU would not have been able to compare plans in a uniform manner across the EU due to a lack of EU baseline standards and indicators.^77^ With the creation of the European Health Union, these previous shortcomings and weak points are to be eliminated by coordinating at EU level. The measures that the EU envisages for preparedness and response planning at the EU level are thus exclusively of a coordinating nature to support the Member States.

### Health Emergency Preparedness and Response Authority (HERA)

As a key element of the European Health Union, the European Commission established a new EU authority, the Health Emergency Preparedness and Response Authority (HERA), in September 2021 to prevent, detect and respond rapidly to health emergencies such as the Covid 19 pandemic.^78^ One of HERA’s main tasks is to anticipate future health threats and potential emergencies by gathering information and building the necessary response capacity.^79^ In the event of an emergency, HERA will ensure the development, production and distribution of medicines, vaccines and other medical countermeasures, such as gloves and masks, that were often lacking in the first phase of the Covid 19 pandemic.^80^ As the EU Commission points out, HERA is a key pillar of the European Health Union and will fill a gap in the EU’s emergency response and preparedness.

Before a health crisis, HERA will work closely with other EU and national health agencies industry and international partners in the “preparedness phase” to improve the EU’s readiness for health emergencies, carry out threat assessments and intelligence gathering, develop models to forecast an outbreak, and support research and innovation for the development of new medical countermeasures, including through Union-wide clinical trial networks and platforms for the rapid sharing of data.^81^

In addition, HERA is also tasked with emergency measures during a health crisis. In the event of a public health emergency at EU level, HERA, under the steer of a high-level Health Crisis Board, can quickly switch to emergency measures such as activating emergency funding, launch mechanisms for monitoring, and undertaking the targeted development, procurement and purchase of medical countermeasures and raw materials.^82^

### Evaluation of the European Health Union

With regard to an analysis of the European Health Union, it should first be noted that the European Health Union with its initiatives, its tasks, the planned measures and in particular its actors, adheres to within the framework of the existing competence structure according to Art. 168 TFEU. Since the competences of the EU are limited in the area of health protection and the EU has no general competence to pursue autonomous health policy, it becomes clear against this background that it is not entitled to take any further-reaching measures for the protection and combating of epidemics and pandemics under Art. 168 TFEU. This also applies to the creation of a European Health Union and all related measures and initiatives.

According to Art. 168 (5) TFEU, the EU may adopt measures, e.g. for early warning and combating of serious cross-border health threats, which it has already done so far.^83^ However, Art. 168 (5) TFEU provides that the EU may not act regarding harmonisation. Consequently, the establishment of a European Health Union in response to the Covid 19 pandemic does not create any new competences and does not give the EU Commission and its agencies any further competences than those supporting, coordinating and complementary competences that have been conferred on it under Art. 168 TFEU. Consequently, also within the framework of the European Health Union, the EU is basically limited to advisory and coordinating measures to support the Member States.

Even within the framework of a European Health Union, the Member States continue to be responsible for appropriate measures on health protection and medical care. The Member States must decide whether further coordination of measures at the EU level should take place in the event of future pandemics and whether or not competences should be transferred to the EU for this purpose. Against this background, the European Health Union represents a further development of existing measures, programmes, agencies and networks, which takes important and essential new steps, but in terms of content and institution remains within the previous competence structure of Art. 168 TFEU.

## Further EU competences after Covid 19?

### Some considerations

In the light of the Covid 19 pandemic, questions about health protection by the EU arise in a new context. In particular, the question can be raised whether the EU – in view of the threat and consequences of the Covid 19 pandemic and future pandemics that may be even more serious and deadlier – should have a greater weight in terms of more coordinated and determined action to combat communicable diseases, especially epidemics and pandemics.^84^

As the EU’s response to the threat, problems and consequences of the Covid 19 pandemic with regard to the European Health Union shows, existing measures can be improved, intensified and made more effective overall at EU level. However, as already analysed, these measures are limited due to the system of competences and the limited competences assigned to the EU in the area of health policy according to Art. 168 TFEU, which are essentially restricted to the areas of coordination and support of the Member States. The measures can therefore in principle only serve to coordinate and complement the policies of the Member States, excluding harmonisation of national laws regulations of the Member States.^85^ The measures taken by the EU in the context of the Covid 19 pandemic have shown that, where the EU institutions had their own competences, they usually acted quickly and in a targeted manner.^86^ Due to the current system of competences in the area of health policy and the limited competences of the EU in this area, faster action in the relevant areas was often not possible.

Instead, the main responsibility for crisis policy in the context of the Covid 19 pandemic lay with the governments of the Member States, which declared their basic willingness to cooperate and take joint measures at the EU level, but also partly showed national interests that did not lead to coordination at the EU level.^87^ During the Covid 19 pandemic, it could be observed that the Member States often acted in an uncoordinated manner, which meant that measures were often only coordinated after relevant events or were not coordinated at all.^88^ Whether this was due to time constraints or political motives is relevant to the question of a further transfer of competence to the EU – but irrelevant with regard to the question of an efficient approach in the event of a pandemic with the potential of a health emergency of international scope. The Covid 19 pandemic therefore makes it clear that the EU still fundamentally has in principle only coordinating competences in the area of health policy and therefore depends on the consensus and participation of the Member States for its measures.^89^

### Competences and indirect legislation

In the light of these observations, the key question is whether the EU should be given further competences to act at EU level in this area, while respecting the principle of subsidiarity.^90^ According to Regulation 851/2004,^91^ the ECDC has a special role to play in the fight against communicable diseases, in particular in the establishment of a European early warning and response system and in monitoring the control of communicable diseases.^92^ Nevertheless, the EU cannot rely on a fundamental competence for independent and Union-wide pandemic control.^93^

An interesting aspect in this context is the importance of the internal market regulations according to Art. 114 TFEU, which have led to harmonisation at the EU level in other areas, such as food, consumer goods and tobacco products. This is associated with the possibility of indirect legislation, which also (co-)regulates aspects of health protection. The EU Commission’s communication on the creation of a European Health Union contains an interesting reference to this.^94^ The EU Commission points out that a strong European Health Union will protect “our way of living, our economies and societies”, since the “economy inevitably suffers” if “public health is danger”.^95^ In this sense, the EU Commission emphasizes that the European Health Union will also contribute to “a more resilient EU internal market and a sustained economic recovery”. In this context, it might seem useful to have an explicit reflection on the EU’s competences in the field of infection control. This would at the same time also clarify questions of indirect legislation in this area via the internal market provisions of Art. 114 TFEU.

### Arguments for an extension of competence

The Covid 19 pandemic has made it clear that the EU basically only has a coordinating competence in the area of health policy and therefore dependents on the consensus and cooperation of the Member States for its measures.^96^ At the same time, however, only a common European strategy can ensure effective measures in dealing with a pandemic.^97^ This means that in some areas, such as vaccine procurement, where there is a discrepancy between the European task and competence, this could be resolved by supplementing existing competences, while observing the criteria of the subsidiarity principle.^98^ It is true, as already explained and analysed, that in some specific areas, such as certain medicinal products and medical devices, the EU already has competence based on Art. 114 and 168 TFEU, which enables it to enact binding legislation for certain medicinal products, such as advanced therapy medicinal products (ATMPs), as well as for medical devices.^99^ Thus, these specific areas are subject to the rules of the internal market.^100^

However, according to the design of Art. 168 TFEU, further EU health protection measures in the context of the Covid 19 pandemic, as well as with regard to future pandemics, are currently excluded. According to Art. 168 (1) TFEU, the objective of health protection at EU level is to ensure a “high level of human health protection”, but only with regard to the definition and implementation of all EU policies and measures, i.e. only to the extent that competences are assigned to the EU. In this respect, “objectives” are not to be equated with “competences”.

Based on the principle of conferral according to Art. 5 TEU, as already examined, the institutions of the EU can only enact legal norms if they are explicitly authorised to do so by primary law. Accordingly, the EU itself cannot establish any competences. The EU only acts within the limits of the competences that the Member States have conferred on it. All competences that are not transferred to the EU under primary law remain with the Member States. For the area relevant in the event of a pandemic, “protection and improvement of human health” according to Art. 6 lit. a TFEU, the EU is only competent according to Art. 2 (5) TFEU “under the conditions laid down in the Treaties” to “carry out actions to support, coordinate or supplement the actions of the Member States, without thereby superseding their competence in these areas”. In view of the fact that the EU can – at least in part – indirectly regulate the health and infection protection law of the Member States via the internal market and the right of free movement, and in view of the indicated problems which are to be solved within the framework of the creation of a European Health Union, it might seem appropriate to reconsider the current allocation of competences to the EU – at least regarding the protection, prevention and response against serious human health risks in case of pandemic – at the political level and, if necessary, to expand it.

### Approach to a solution and questions for discussion

One approach to a solution for further discussion could be to expand the list of exceptions to the prohibition of harmonisation. In principle, the EU only has the aforementioned coordinating competences to support the Member States. However, Art. 168 (4) TFEU provides for exceptions to the prohibition of harmonisation in the areas explicitly listed therein, which concern measures to meet common safety concerns.^101^ In these narrowly defined areas, the EU, as already explained, has shared legislative competence.^102^

Since the areas of prevention, control and combating of infectious diseases, in particular pandemics, could be attributed to the common safety concerns of the Member States, consideration could be given to expanding the exceptions under Art. 168 (4) TFEU with a further narrowly defined fourth case of “measures to prevent, control and combat communicable diseases with pandemic potential”.^103^ This would not only mean that the prohibition on harmonisation laid down in Art. 168 (5) TFEU would remain in place, as the addition refers to an exceptional case of this prohibition. It would also allow support measures to protect and improve human health and, in particular, to combat widespread serious cross-border diseases, measures to monitor, report at an early stage and combat serious cross-border threats to health as well as measures as also provided for in Art. 168 (5) TFEU.

## Results and conclusion

An analysis of health protection at the level of the EU must first be based on the system of competences pursuant to Art. 168 TFEU, according to which, in the area of health policy and the protection of public health, the EU has, in principle, only been assigned coordinating competences to support the Member States. The principle applies that the Member States themselves determine their national health policy, the organisation and financing of healthcare and, in principle, also the protection of public health within their national territory.

Nevertheless, certain competences have been transferred to the EU under Art. 168 TFEU. On the basis of these competences, the EU has established the Network for the Epidemiological Surveillance and Control of Communicable Diseases, the Health Security Committee (HSC), an Early Warning Response System (EWRS) and the European Centre for Disease Prevention and Control (ECDC).

Against this background, however, it should be noted that pandemics – as the Covid 19 pandemic has made clear – are a genuinely global problem and precisely not exclusively a European matter. By their nature and definition, and also from a legal perspective, pandemics are no more an EU problem than they are an exclusive problem for individual states. Rather, it is a global problem that primarily requires action by states to prevent, control and combat it at the global level.

However, due to the internal market with the fundamental freedoms, in particular the free movement of persons, coordination at EU level is required. For the successful and reliable prevention, control and combating of cross-border infectious diseases, immediate, planned and coordinated measures are therefore indispensable not only at the national level of the Member States, but especially also at the EU level. This necessity arises in particular in an area without internal frontiers with free movement of persons, services and goods. Even before the Covid 19 pandemic, this necessity was recognised and addressed in particular by the Health Security Committee (HSC) and the European Centre for Disease Prevention and Control (ECDC).

A possibility for a further transfer of competence to the EU – while largely respecting the existing competence structure according to Art. 168 TFEU and taking into account the prohibition of harmonisation according to Art. 168 (5) p. 1 TFEU – would be to expand the list of exceptions according to Art. 168 (4) TFEU with a further exception regarding “measures to protect public health in the case of infectious diseases with pandemic potential in the EU”. This would not only have the advantage that no competences outside of an infectious disease with pandemic potential would be transferred to the EU. It would also ensure that the transfer of competence would be included in a list of exceptions and that it would thus, in principle, be interpreted narrowly.

The Covid 19 pandemic can therefore be seen as a historical turning point. In view of the European Health Union and the competences of the EU, however, it does not (yet) represent a fundamental caesura. Nevertheless, on the basis of the European Health Union, further developments and adjustments of health protection at the EU level – and perhaps also a further transfer of (narrowly defined) competences to the EU – could take place.

## Footnotes

1 Covid 19 (corona virus disease 19) refers to a new and hitherto unknown infectious disease which is clearly different from other diseases caused by coronaviruses, such as SARS and MERS, because the virus spreads rapidly and outbreaks increase exponentially. It should be noted that the spelling is not uniform and sometimes e.g. Covid-19, Covid 19 or COVID 19 is also used in official documents. For reasons of uniform use, the term “Covid 19” is used in this article with the exception of word quotations. On the background of the coronavirus, see *Vogel* [50], p. 1 et seq.

2 On the socio-economic consequences of the Covid 19 pandemic, see *Bergmann* [3], p. 45; *Iskan et al.* [19], p. 58 et seq.; *Mögele* [32]; *Repasi* [37]; *Seitz et al.* [45].

3 On the impact of the Covid 19 pandemic, which is more than just a global emergency and an international health crisis, see United Nations [49], p. 8 et seq.

4 *Iskan* [20], p. 1; *Siegenthaler et al.* [46].

5 On the ordered restrictions of freedom due to the Covid 19 pandemic and questions of a more general constitutional discourse, see *Hase* [14]; *Hase* [16]; *Heinig et al.* [17]; *Kersten et al.* [23]. On the democratic legitimacy of state measures in the event of a pandemic, see *Klafki* [25], p. 215 et seq.

6 A Public Health Emergency of International Concern (PHEIC) is a formal declaration by the WHO in the case of an extraordinary event which is determined to constitute a public health risk to other states through the international spread of disease and to potentially require a coordinated international response. Thus, a PHEIC constitutes a situation that is serious, sudden, unusual, or unexpected and which carries implications for public health beyond the affected state’s national border. As such it may require immediate international action and member states have an obligation to respond promptly to a PHEIC and undertake appropriate actions.

7 The International Health Regulations (IHR) of the WHO were first adopted by the World Health Assembly in 1969 and revised in 2005. It is an international legal treaty which empowers the WHO to establish and act as a global surveillance system.

8 The health systems for all EU Member States plus Iceland and Norway were examined by the Organisation for Economic Co-operation and Development (OECD) and the European Observatory on Health Systems and Policies (Observatory). For an overview, see EU Commission, Country Health Profiles [15].

9 The health care systems of the EU Member States can basically be divided into the two organisational forms of tax-financed national health care on the one hand and systems financed by social insurance on the other, although the two systems are now partly converging.

10 The EU Commission has compiled an overview of the national portals of the EU Member States with regard to the measures taken during the Covid 19 pandemic, see EU Commission, Public Health [16].

11 Council of the European Union, Council Conclusions on “Common values and principles in European Union Health Systems” [2006] OJ C 146/1, p. 1.

12 Council of the European Union [2006] OJ C 146/1, p. 1.

13 Treaty on European Union, [1992] OJ C 191/1 (Treaty of Maastricht).

14 For further information on the anchoring of health protection at EU level and its development in the Treaties, reference is made to the Fact Sheets on the European Union, EU Parliament. Available at: https://www.europarl.europa.eu/factsheets/de/sheet/49/offentliche-gesundheit.

15 Treaty of Amsterdam amending the Treaty on European Union, the Treaties establishing the European Communities and certain related acts, [1997] OJ C 340/1 (Treaty of Amsterdam).

16 Thus, Art. 129 (1) of the Treaty of Amsterdam states: “A high level of human health protection shall be ensured in the definition and implementation of all Community policies and activities. Community action, which shall complement national policies, shall be directed towards improving public health, preventing human illness and diseases, and obviating sources of danger to human health. Such action shall cover the fight against the major health scourges, by promoting research into their causes, their transmission and their prevention, as well as health information and education.”

17 Treaty of Lisbon amending the Treaty on European Union and the Treaty establishing the European Community, [2007] OJ C 306/1 (Treaty of Lisbon).

18 For an overview of the developmental steps towards an EU health policy, see *Kment* [26]; *Pitschas* [34]; *von Schwanenflügel* [52], p. 10 et seq.; *von Schwanenflügel* [51].

19 Treaty on the Functioning of the European Union, consolidated version, [2008] OJ C 115/13, p. 47 (TFEU).

20 On the history and scope of EU health law and policy, see *Guy et al.* [13], p. 17; *Hanika* [14]; *Sindbjerg* [47], p. 36.

21 See *Leidenmühler* [28], p. 398.

22 The definition of the title is not uniform in the different language versions of the TFEU. The English version, for example, speaks of “public health” while the German version speaks of “Gesundheitswesen” which means “health care”.

23 See *Schmidt am Busch* [41], Art. 168 TFEU, para. 9. The title of Title XIV of the TFEU “Public Health” is in this respect not precise and in other language versions partly broader.

24 See *Lurger* [29], Art. 168, para. 9.

25 Charter of Fundamental Rights of the European Union, [2000] OJ C 364/1, p. 1 (European Charter of Fundamental Rights, CFR).

26 See the explanations of the Praesidium of the European Convention, [2017] OJ C 303/17, p. 1, 27: Art. 35 CFR “corresponds to Art.168 (1)”. The right under Art. 35 CFR is concretised by Art. 168 TFEU to a certain extent, whereby it should be noted that Art. 168 TFEU is the “older provision”, as it was already enshrined in primary law before the CRF entered into force.

27 For more details, see *Sander* [39]; *Streinz* [48], Art. 168 TFEU, para. 17.

28 On the principle of conferral according to Art. 5 (1) TEU, see *Breitenmoser et al.* [6], p. 182; *Buschmann et al.* [8]; *Calliess* [9]; *Winter* [54].

29 See *Breitenmoser et al.* [6], p. 182.

30 On the subsidiarity principle in Art. 5 TEU and its control, see *Bickenbach* [4]; *Ritzer et al.* [38].

31 For an overview of the individual types of competence, see *Kingreen* [24], Art. 168 TFEU, para. 3.

32 See *Schmidt am Busch* [41], Art. 168, para. 8.

33 See *Seitz* [44].

34 *Schmidt am Busch* [41], Art. 168, para. 11.

35 See *Maass et al.* [30]; *Purnhagen* [35].

36 See *Kingreen* [24], Art. 168 TFEU, para. 1.

37 On the shared legislative competences in the exceptional areas according to Art. 168 TFEU, see *Calliess* [9]. Other exceptional areas under Art.168 (4) TFEU in which the EU has legislative competence include “measures setting high standards of quality and safety of organs and substances of human origin, blood and blood derivatives” (lit. a) and “measures in the veterinary and phytosanitary fields which have as their direct objective the protection of public health” (lit. b).

38 See *Voß* [53], p. 152.

39 See *Schnell et al.* [42], p. 283.

40 See *Kamann et al.* [22], with examples of exceptions to the competence of Member States under Art. 167 (7) TFEU.

41 On the distribution of competences between the EU and its Member States see *Kurscheid et al.* [27], p. 158.

42 See *Schulte* [43], in the context of the European benefit assessment of medicinal products and medical devices with regard to questions of cost-effectiveness, which fall within the scope of reimbursement conditions for healthcare products.

43 See *Purnhagen* [35]; *Schmidt et al.* [40].

44 On the measures of the EU with regard to the Covid 19 pandemic in detail, see *Alemanno* [1]; *Gehler* [12], p. 67 (81).

45 See *Gehler* [12], p.67 (81); *Müller* [33], p. 59 (67).

46 For an overview of EU measures during the Covid 19 pandemic, see EU Commission, Corona Crisis Response. Available at: https://ec.europa.eu/info/live-work-travel-eu/coronavirus-response.de.

47 Decision No 2119/98/EC of the European Parliament and of the Council of 24.9.1998 setting up a network for the epidemiological surveillance and control of communicable diseases in the Community, [1998] OJ L 268 p. 1.

48 Decision No. 2119/98/EC, Art. 2.

49 Decision No 1082/2013/EU of the European Parliament and of the Council of 22.10.2013 on serious cross-border threats to health and repealing Decision 2119/98/EC, [2013] OJ L 293 p. 1.

50 Health Security Committee (HSC), http://ec.europa.eu/health/preparedness_response/risk_management/index_de.htm.

51 See *Kersten et al.* [23], p. 137; *Mögele* [32].

52 Regulation (EC) No 851/2004 of the European Parliament and of the Council of 21.4.2004 establishing a European Centre for disease prevention and control, [2004] OJ L 142, p. 1.

53 European Centre for Disease Prevention and Control (ECDC), http://ecdc.europa.eu/en/Pages/home.aspx.

54 Regulation (EC) No 851/2004, p. 1.

55 See *Deruelle et al.* [11].

56 *Deruelle et al.* [11].

57 See *Jordana et al.* [21].

58 See *Purnhagen et al.* [36], p. 297.

59 See *Deruelle et al.* [11].

60 EU Commission, European Health Union: Protecting the health of Europeans and collectively responding for cross-border health crises, https://ec.europa.eu/info/strategy/priorities-2019-2024/promoting-our-european-way-life/european-health-union_de.

61 EU Commission, European Health Union: Protecting the health of Europeans and collectively responding for cross-border health crises, https://ec.europa.eu/info/strategy/priorities-2019-2024/promoting-our-european-way-life/european-health-union_de.

62 Communication from the Commission to the European Parliament, the Council, the European Economic and Social Committee and the Committee of the Regions of 11.11.2020 on “Building a European Health Union: Reinforcing the EU’s resilience for cross-border health threats”, COM (2020) 724 final, https://eur-lex.europa.eu/legal-content/EN/TXT/PDF/?uri=CELEX:52020DC0724&from=EN.

63 On the European Health Union, see *Ambrosius et al.* [2]; *Henke* [18]; *Müller* [33]. On the challenges of care in times of crisis and the coordination of measures between the EU and EU Member States, see *Bossung et al.* [5], p. 64; *Brooks et al.* [7], p. 1057.

64 *Von der Leyen, Ursula*, President of the European Commission, at the World Health Summit on 25.10.2020, https://ec.europa.eu/info/strategy/priorities-2019-2024/promoting-our-european-way-life/european-health-union_de.

65 European Council, Joint statement of the Members of the European Council of 26.3.2020, https://www.consilium.europa.eu/de/press/press-releases/2020/03/26/joint-statement-of-the-members-of-the-european-council-26-march-2020. For an overview see *Müller* [33].

66 European Parliament, Press release: Parliament wants a European Health Union of 10.7.2020, https://www.europarl.europa.eu/news/en/press-room/20200710IPR83101/parliament-wants-a-european-health-union.

67 EU Commission, European Health Union: Protecting the health of Europeans and collectively responding to cross-border health crises, https://ec.europa.eu/info/strategy/priorities-2019-2024/promoting-our-european-way-life/european-health-union_de.

68 EU Commission, European Health Union: Protecting the health of Europeans and collectively responding to cross-border health crises.

69 Communication, COM (2020) 724 final, 2.

70 Communication, COM (2020) 724 final, 2.

71 Communication, COM (2020) 724 final, 2.

72 EU Commission, European Health Union: Protecting the health of Europeans and collectively responding to cross-border health crises.

73 For an overview of the planned measures within the framework of the European Health Union, see Communication from the Commission to the European Parliament, the Council, the European Economic and Social Committee and the Committee of the Regions of 11.11.2020 on “Building a European Health Union: Reinforcing the EU’s resilience for cross-border health threats”, COM (2020) 724 final, p. 9.

74 See EU Commission, European Health Union: Protecting the health of Europeans and collectively responding to cross-border health crises.

75 Communication from the Commission to the European Parliament, the Council, the European Economic and Social Committee and the Committee of the Regions of 11.11. 2020 on “Building a European Health Union: Reinforcing the EU’s resilience for cross-border health threats”, COM (2020) 724 final, p. 1 (12).

76 Communication from the Commission, COM (2020) 724 final, p. 1 (12).

77 Communication from the Commission, COM (2020) 724 final, p. 1 (12).

78 EU Commission, Decision of 16.9.2021 establishing the Health Emergency Preparedness and Response Authority (HERA), C(2021) 6712 final. On the Health Emergency Preparedness and Response Authority (HERA), see https://ec.europa.eu/commission/presscorner/detail/de/ip_21_4672.

79 Communication, COM (2020) 724 final, 2.

80 See EU Commission, European Health Emergeny Preparedness and Response Authority (HERA): Getting ready for future health emergencies, see https://health.ec.europa.eu/latest-updates/european-health-emergency-preparedness-and-response-authority-hera-getting-ready-future-health-2021-09-16-0_en.

81 EU Commission, European Health Emergeny preparedness and Response Authority (HERA).

82 EU Commission, European Health Emergeny preparedness and Response Authority (HERA).

83 See for example Decision No. 1082/2013/EU on serious cross-border health threats.

84 See *Calliess* [9]; *Müller* [33]; *Seitz* [44].

85 See *Mögele* [32]; *Seitz* [44].

86 See *Müller* [33], p. 67.

87 See *Müller* [33], p. 67.

88 *Müller* [33], p. 67.

89 See also *Calliess* [9], who points out in this context that political actors at the EU level tend to promise too much to EU citizens, but that the EU institutions are not in a position to deliver due to their competences.

90 On the current legal starting position, see *Calliess* [9]; *Mögele* [32]; *Seitz* [44].

91 Regulation (EC) No 851/2004 of the European Parliament and of the Council of 21.4. 2004 establishing a European Centre for disease prevention and control, [2004] OJ L 142, p. 1.

92 See *Seitz* [44].

93 See *Calliess* [10].

94 Communication from the Commission, COM (2020) 724 final, p. 2.

95 Communication from the Commission, COM (2020) 724 final, p. 2/3.

96 See *Mögele* [32].

97 See *Calliess* [9].

98 *Calliess* [9].

99 See *Seitz* [44].

100 In the EU, the “shared competence” rule applies to medicinal products, which has its legal basis in Art. 4 (2) TFEU). Member States, however, retain exclusive competence for the distribution of medicinal products, for the organisation and delivery of health services pursuant to Art. 168 (7) TFEU.

101 The literature points to a certain erosion of state sovereignty with regard to Art. 168 (4) and (5) TFEU, see *Marino* [31], p. 1 (29).

102 See *Marino* [31], p. 1 (29).

103 See as well *Calliess* [9].
